# Effects of four kinds of electromagnetic fields (EMF) with different frequency spectrum bands on ovariectomized osteoporosis in mice

**DOI:** 10.1038/s41598-017-00668-w

**Published:** 2017-04-03

**Authors:** Tao Lei, Feijiang Li, Zhuowen Liang, Chi Tang, Kangning Xie, Pan Wang, Xu Dong, Shuai Shan, Juan Liu, Qiaoling Xu, Erping Luo, Guanghao Shen

**Affiliations:** 1 School of Biomedical Engineering, Fourth Military Medical University, 17 West Changle Road, Xi’an, China; 2 Institute of Orthopaedics, Xijing hospital, Fourth Military Medical University, Xi’an, China; 3 School of Nursing, Fourth Military Medical University, 17 West Changle Road, Xi’an, China

## Abstract

Electromagnetic fields (EMF) was considered as a non-invasive modality for treatment of osteoporosis while the effects were diverse with EMF parameters in time domain. In present study, we extended analysis of EMF characteristics from time domain to frequency domain, aiming to investigate effects of four kinds of EMF (LP (1–100 Hz), BP (100–3,000 Hz), HP (3,000–50,000 Hz) and AP (1–50,000 Hz)) on ovariectomized (OVX) osteoporosis (OP) in mice. Forty-eight 3-month-old female BALB/c mice were equally assigned to Sham, OVX, OVX + LP, OVX + BP, OVX + HP and OVX + AP groups (n = 8). After 8-week exposure (3 h/day), LP and BP significantly increased serum bone formation markers and osteogenesis-related gene expressions compared with OVX. Bedsides, LP and BP also slightly increased bone resorption activity compared with OVX, evidenced by increased RANKL/OPG ratio. HP sharply decreased serum bone formation and resporption markers and osteogenesis and osteoclastogenesis related gene expressions compared with OVX. AP had accumulative effects of LP, BP and HP, which significantly increased bone formation and decreased bone resporption activity compared with OVX. As a result, LP, BP and HP exposure did not later deterioration of bone mass, microarchitecture and mechanical strength in OVX mice with OP. However, AP stimulation attenuated OVX-induced bone loss.

## Introduction

Osteoporosis (OP) is a serious health problem that is especially related to aging in postmenopausal women, which is characterized by skeletal fragility and microarchitectural deterioration^1, 2^. By 2050, the worldwide incidence of hip fracture in women is expected to increase by 310%, mainly due to the ageing of the worldwide population^3^. Bone remodeling is a continuous process between bone resorption (activity of osteoclasts (OCs)) and formation (activity of osteoblasts (OBs)). The absence of estrogen induced by the menopause increases the formation and the activity of OCs, which play key roles in bone loss, and OCs ultimately increase the risk of menopausal OP^4^. Therefore, inhibiting the formation and function of OCs and enhance the formation and function of OBs are important therapeutic strategies. Various pharmacological treatments are available for postmenopausal OP, such as estrogen replacement therapy, bisphosphonate and calcitonin^5, 6^. However, side effects occur when these are used excessively, such as breast cancer or endometrial cancer^6, 7^. An alternative therapy which is worthy of consideration in the treatment of OP is EMF, which has been investigated as a noninvasive alternative method^8–11^. It has been reported that EMF can increase bone mineral density (BMD) in OP patients^12^, prevent bone loss in OVX induced OP *in vivo* ^9, 13^, and affect bone metabolism *in vitro* ^14^. However, the efficacy of this modality remains uncertain currently^15, 16^. Moreover, the studies also showed us the conflicting results^16, 17^. The different results are usually explained by different intensity, frequency, waveform and duration of EMF. Therefore, can we discard these numerous and complicated parameters to find other substitutes to accurately and simply describe the EMF, aiming to increase the repeatability and reproducibility of the experiments? There is a report implied that the dominant influence of EMF in OBs is not related to variables of EMF those expressed in the time domain, extension of the analysis to EMF characteristics expressed in the frequency domain should be encouraged^18^. Therefore, focusing on the frequency spectrum of EMF might facilitate to investigate the possible mechanisms of EMF on bone healing. There are good evidences that low frequency EMF can produce resonance interactions that influence ion movements through membrane channels and other biological phenomena when the frequency of EMF matches cyclotron resonance frequencies of critical ions^19–21^. Investigators have suggested that the physical mechanism underlying these effects is ion cyclotron resonance (ICR)^22–24^. According to ICR model, the resonant frequencies of many biologically important ions, such as Na^+^, K^+^ and Ca^2+^, are intermittent frequency points and fall within 1–100 Hz^23, 25^. Aparting from the fundamental frequency of resonant frequencies, when the frequency of EMF is equal to higher harmonics of the cyclotron frequencies, the biological resonant effectiveness might also be attained^26, 27^. Moreover, these higher harmonics of the cyclotron frequencies of the biologically relevant ions is blow 3,000 Hz^24^. In addition, high frequency EMF is also capable of inducing osteogenic differentiation of osteoprogenitor cells^28^. Therefore, we designed four kinds of EMF with different frequency spectrum bands (1–100 Hz, 100–3,000 Hz, 3,000–50,000 Hz and 1–50,000 Hz), among which 1–100 Hz and 100–3,000 Hz are designated as ICR frequency bands.

Some investigators have demonstrated that an OVX mouse could be used as an experimental animal model of postmenopausal OP^29^. This study aimed to investigate the effects of four kinds of EMF with different frequency bands on bone mass, microarchitecture and strength in OVX mice with OP.

## Materials and Methods

### EMF exposure system

**Figure 1:**
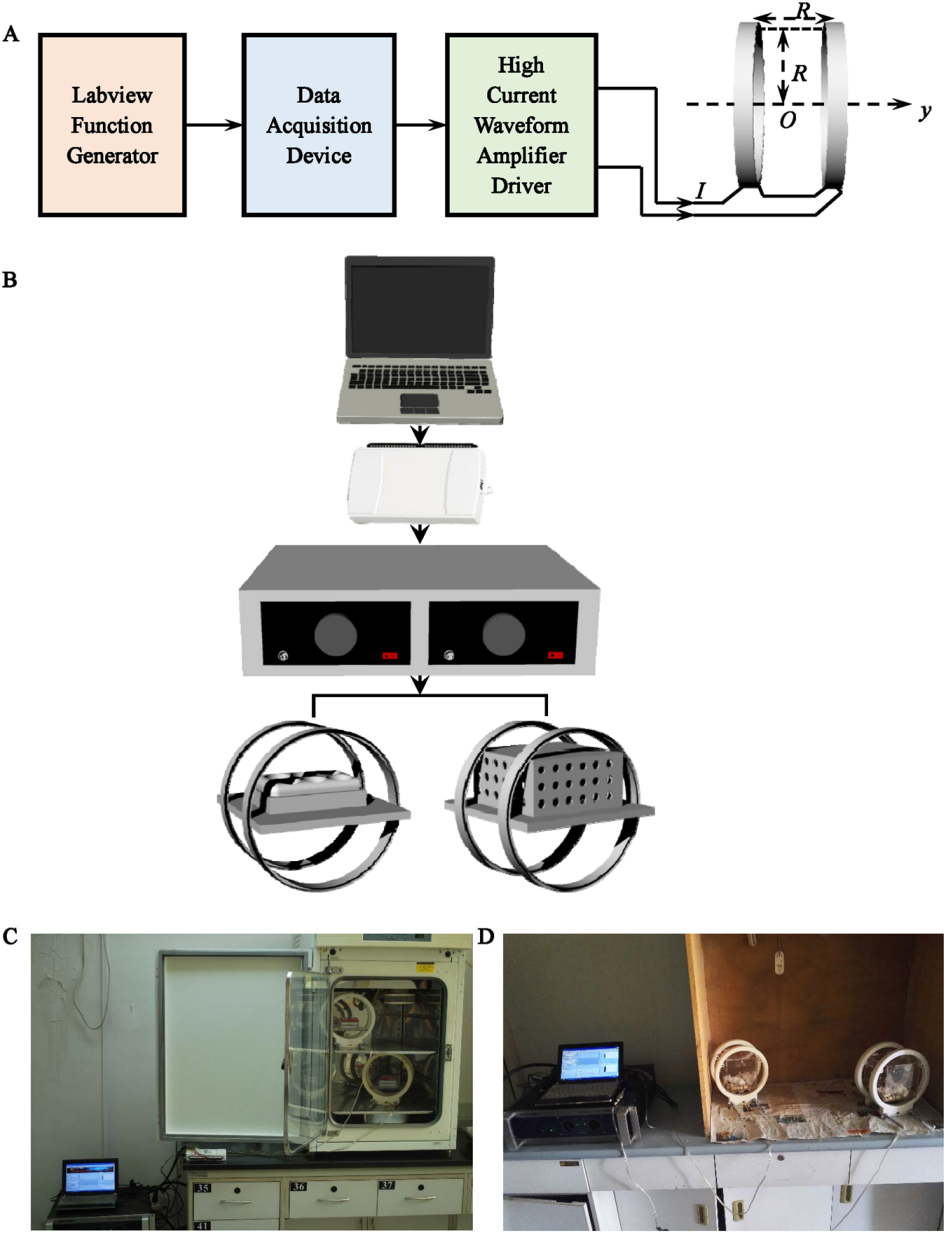
Representation of the system used to generate EMF. (**A**) The principle block diagram of EMF system. The device consists of four main parts: Labview software, multifunction data acquisition device, power amplifier and Helmholtz coils. (**B**) The simulated effect diagram of the system. (**C**) The physical photo of EMF system in the experiment.

**Figure 2:**
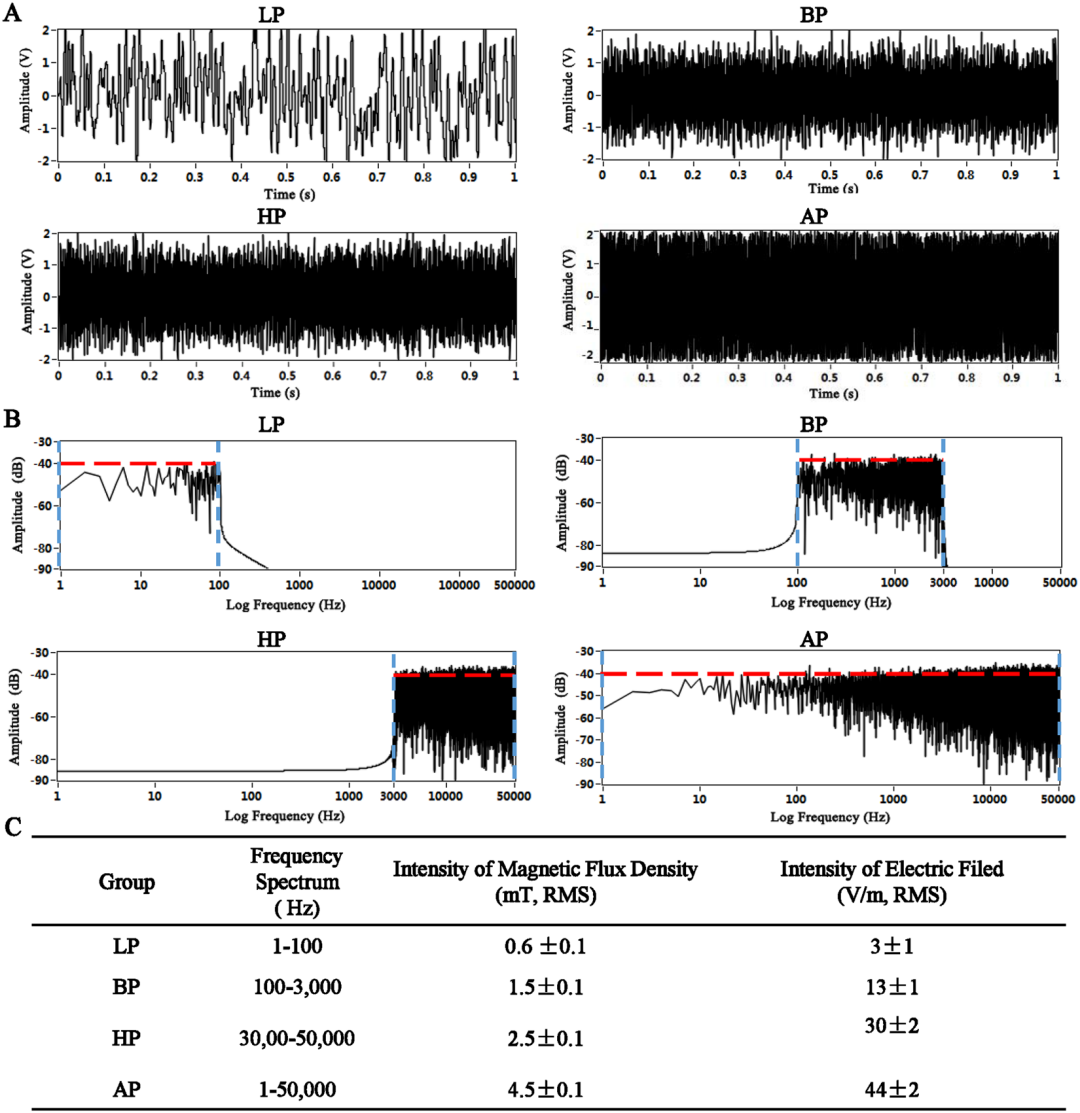
Illustration of four kinds of EMF waveform signals in time and frequency domain and the measurement results of magnetic flux density and electric field strength of four kinds of EMF. (**A**) Four kinds of EMF waveform signals in time domain. (**B**) Four kinds of EMF waveform signals in time domain. (**C**) Measurement results of magnetic flux density and electric field strength of four kinds of EMF by using a Gaussmeter and an electric field tester.

The EMF exposure system used to stimulate cells was homemade, which was consisted of four parts: Labview software, multifunction data acquisition device (NI USB-6211), power amplifier (XP9900S, Huamei, China) and Helmholtz coils (Fig. 1). The EMF signal was programed by the Labview software. Four kinds of EMF signals were utilized in our study (Fig. 2A, time domain), which were generated from uniform white noise (a random signal with constant power spectral density across all the frequencies (−∞ to +∞)). By filtering with four kinds of signal filters, the open circuit voltage of EMF were low pass signal filtered at 1–100 Hz (LP), band pass signal filtered at 100–3,000 Hz (BP), high pass signal filtered at 3,000–50,000 Hz (HP) and all pass signal filtered at 1–50,000 Hz (AP) (Fig. 2B, frequency domain). Each frequency component of these EMF had the same amplitude (−40 dB). USB-6211 has two analog outputs (16 bit, 250 kS/s), which realizes converting digital filtered EMF signals to analog signals. XP9900s with two output channels amplifies the EMF signals to drive the Helmholtz coils, and its output power is 1,400 W, the impedance is 4–8 Ω, and the frequency response range is 1 Hz–55 KHz. The Helmholtz coils were consisted of two similar coils with radius R and N wire windings (enamel copper wire, 0.6 mm in diameter) placed in the same distance R, where R = 10 cm, N = 80. The coils were connected serially, thus, the current through the coils flows in the same direction, and it produces a region with a nearly uniform magnetic field (Supporting Fig. S1), which was demonstrated by the finite element engineering software called COMSOL Multiphysics (v4.3 COMSOL AB, Burlington, MA, USA). The homemade porous cubic plastic cages containing mice without anesthesia were put in the center of the Helmholtz coils and cages were supported by stands to let the activities of mice restrict on the center plane of the Helmholtz coils which had higher intensity and better uniformity of magnetic flux density (Fig. 1B,D). Moreover, whole body exposure to EMF for mice without anesthesia was applied three hours every day. The magnetic flux density and electric field strength were measured by using a Gaussmeter (Model 455 DMP Gaussmeter, Lake Shore Cryotronics, USA) and an electric field tester (Modle GM3120, Benetech, China), and the measurement results were listed in Fig. 2C. In case of interference of temperature brought by coils, the CO_2_ incubator was monitored by the incubator’s temperature sensor, and the temperature was in the range of 37 ± 0.2 °C throughout the experiments.

### OVX model and exposure protocol

Forty-eight 3-month-old female BALB/c mice weighting 31.7 ± 1.2 g were provided by Animal Center of the Fourth Military Medical University and housed in a room (Animal Center of the Fourth Military Medical University, Xi’an, China). Mice were housed in controlled temperature (23 ± 1 °C), relative humidity (50~60%) and alternately light-dark cycle (12 h/12 h), with access to standard pellet and clean water. After one week of acclimatization, all mice were subjected either a sham surgery or bilateral ovariectomy as described previously^30^. All mice were anesthetized with an i.p. injection of pentobarbital (50 mg/kg). The sham surgery for eight mice involved the exposure of the ovaries with extraction of the surrounding fatty tissue of bilateral ovaries, leaving the ovaries intact, whereas bilateral ovariectomy for 40 mice involved the full removal of the both the left and right ovaries. After the surgery, penicillin was injected i.m. to each mouse to prevent infection once daily for two days. Mice were allowed to recover from surgery for one week prior to experiments, and then all mice were randomly divided into the following six groups (eight mice in each group): sham-operated control group (Sham), ovariectomy group (OVX), OVX + LP exposure group, OVX + BP exposure group, OVX + HP exposure group and OVX + AP exposure group. OVX + LP, OVX + BP, OVX + HP and OVX + AP groups were exposed to four kinds of EMF respectively for 3 h/day, 7 days/week, for 8 weeks. The current study was performed in adherence to the National Institutes of Health guidelines for the use of experimental animals, and all animal protocols were approved by the Committee for Ethical Use of Experimental Animals of Fourth Military Medical University^31, 32^.

**Figure 3:**
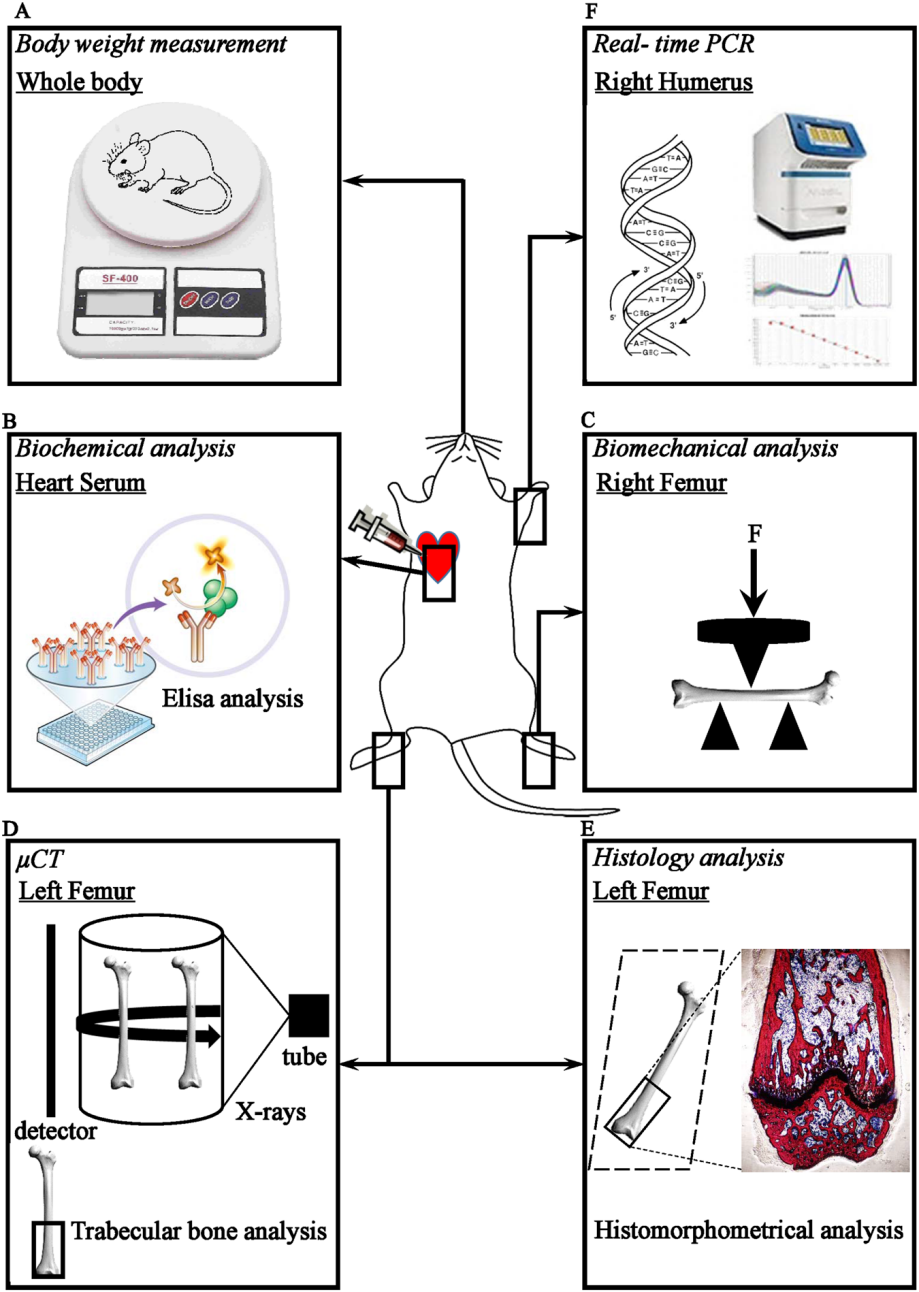
The integral flow chart of *in vivo* experiment. (**A**) Body weight measurement. (**B**) Biochemical analysis of serum. (**C**) Biomechanical examination of right femur. (**D**) µCT analysis of left femur. (**E**) Histology and histomorphometry of analysis of left femur. (**F**) Real-time PCR of right humerus.

The body weights were recorded weekly, at the end of 8-week exposure period, mice were sacrificed by cervical dislocation after anesthesia. Biochemical analysis of serum, biomechanical examination of right femur, µCT and histological analysis of left femur and real-time PCR of right humerus were employed in our study, and the integral experiment flow was showed in Fig. 3.

### Biochemical analysis of serum

After 8-week EMF exposure, all mice were killed by cervical dislocation after anesthesia. Blood samples were collected from all mice, kept still for 1 h at room temperature and centrifuged at 2, 000 × g for 20 min at 4 °C and stored at −80 °C. Serum markers for bone formation including bone specific alkaline phosphatase (BALP), serum osteocalcin (OCN), osteoprotegerin (OPG) and N-terminal propeptide of type I procollagen (P1NP) and bone resorption markers including tartrate-resistant acid phosphatase 5b (TRAP-5b) and C-terminal crosslinked telopeptides of type I collagen (CTX-I) were detected using enzyme-linked immunosorbent assay (ELISA) kit (Westang Biological Technology Co., Ltd, Shanghai, China) according to the protocols provided by manufactures.

### Biomechanical examination

The right femurs were selected for three-point bending tests using Enduratec ELF 3220 mechanical testing machine (Bose Corp., Minnetonka, MN) after the soft tissues, skin and muscles were removed from each femur. Each femur was placed with its anterior surface facing upward on two lower support bars 8 mm apart, and the loading bar was positioned at the center of the femur. A press head was subsequently activated to squeeze the center of shaft in bones until fracture occurred. The compressive loading speed was 0.02 mm/s during the testing time. Data was automatically recorded by the material testing device. The biomechanical properties evaluated were the maximum load [a measure of the maximum force that the sample femur withstood before fracture (N)], bending stiffness [the slope on the linear portion of the load-deformation curve related to the bone’s flexural rigidity (N/mm)], energy absorption [area under the load-deformation curve representing the amount of energy absorbed by the sample femur until breakage (N × mm)] and elastic modulus [a measure of the sample femur’s resistance to being deformed elastically when a force is applied to it] which was calculated according to the equation:

1 $$E=F{L}^{3}/48dI,$$

where *F* is the maximum load, *L* is the distance between supporting points on which the bone rests, *d* is the displacement, *L* is the cross-sectional area moment of inertia of the sample femur^33^.

### µCT

The left femur of each mouse was fixed by immersion in 4% paraformaldehyde for 72 h, then the femurs were imaged with a Micro-CT (eXplore Locus SP, GE Healthcare, Canada). The basic scanning parameters were set as the following: voltage 80 kV, current 80 µA, exposure time 3000 ms, total rotation angle 360°, and rotation angle of increment 0.5°. The scanning resolution was 8 µm/slice. 2D and 3D images were obtained for visualization and display. A volume of interest (VOI) that was 1.0 mm long was selected for the analysis of trabecular bone microarchitecture. The VOI started at a distance of 0.5 mm from the lowest end of the growth plate of the distal femur and extended to the proximal end with a distance of 1.0 mm, which excluded all the primary spongiosa and only contained the second spongiosa. The structural parameters of trabecular bone were analyzed using MicroView software (GE Healthcare, Bio-Sciences). The trabecular bone parameters, including trabecular bone mineral density (BMD, mg/cm^3^), trabecular number/thickness/separation (Tb.N (1/mm), Tb.Th (µm) and Tb.Sp (µm) respectively), trabecular bone volume/tissue volume (BV/TV, %), bone surface/bone volume (BS/BV, 1/mm), connectivity density (Conn.D, 1/mm^3^) and structure model indices (SMI) of femurs were calculated. The mid-diaphysis of the femur was analyzed for the evaluation of cortical indices. The VOI for the cortical analysis was selected as a region with 1 mm (0.5 mm on the either side along the midpoint of the femur). The cortical thickness (Ct.Th), cortical area (Ct.Ar), total cross-sectional area inside the periosteal envelope (Tt.Ar) and cortical area fraction (Ct.Ar/Tt.Ar) were quantitatively analyzed by the MicroView software.

### Histology and histomorphometry of trabecular bone

The left femurs were processed without decalcification after scanning with μCT. They were cast directly in methyl methacrylate and representative sections were prepared using a diamond saw and ground to a thickness of 3 mm. Histologic slides were prepared and stained with Van Gieson stains. A commercial image analysis software Image Pro Plus 6.0 (Media Cybernetics Inc., Bethesda, MD, USA) was used to carry histomorphometrical analysis. 0.5 mm proximal to the growth plate, an area of interest (AOI) that was 2 mm long was selected for the analysis of trabecular bone microarchitecture. Three semi-automatically measured values, namely, trabecular area (Tb.Ar), tissue area (T.Ar) and trabecular perimeter (Tb.Pm) were obtained^34^. Then, the BV/TV, Tb.N, Tb.Th and Tb.Sp were calculated with the equations (2–5)^35^:

2 $$BV/TV=100\times Tb.Ar/T.Ar( \% )$$

3 $$Tb.N=(1.199/2)\times (Tb.Pm/T.Ar)(n/mm)$$

4 $$Tb.Th=(2000/1.199)\times (Tb.Ar/Tb.Pm)(\mu m)$$

5 $$Tb.Sp=(2000/1.199)\times (T.Ar-Tb.Ar)/Tb.Pm(\mu m)$$

### Real-time PCR

After animal scarification, fresh right humerus of each mouse was harvested and cleaned with cold PBS. After removal of bone marrow, bone samples were immediately crushed into powder in a mortar containing liquid nitrogen using a pestle. Total RNA was extracted using TRIzol reagent (Invitrogen, Carlsbad, CA, USA). cDNA was synthesized from the mRNA using the PrimeScript^TM^ RT Master Mix (TaKaRa, Dalian, China). The expression levels of osteogenesis and osteoclastogenesis related genes, including alkaline phosphatase (ALP), bone morphogenetic protein-2 (BMP-2), type 1 collagen (COL-1), osteocalcin (OCN), osterix transcription factors (OSX), runt-related transcription factor 2 (Runx2), Wnt1, β-catenin, low-density lipoprotein receptor-related protein 5 (LRP5), osteoprotegerin (OPG), the receptor activator of nuclear factor-kappa B ligand (RANKL) and the receptor activator of nuclear factor-kappa B (RANK) were quantified using real-time PCR, and their primers used were listed in Table 1. Then, the real-time PCR using a SYBR Premix Ex Taq^TM^ II reagent kit (TaKaRa, Dalian, China) was performed using the CFX96 Touch^TM^ Real-time PCR detection system (Bio-Rad, Hercules, CA, USA). GAPDH was used as a housekeeping gene for normalization. All experiments were repeated at least three times. The relative change in gene expression was analyzed by 2^−∆∆CT^ method^36^.

**Table 1:** The sequence of primers used in the present study for real-time PCR.

Gene	Primer	Primer(5′-3′)	Product Length (bp)
ALP	F	GCAGTATGAATTGAATCGGAACAAC	192
	R	ATGGCCTGGTCCATCTCCAC	
BMP-2	F	TGACTGGATCGTGGCACCTC	112
	R	CAGAGTCTGCACTATGGCATGGTTA	
COL-1	F	GACATGTTCAGCTTTGTGGACCTC	119
	R	GGGACCCTTAGGCCATTGTGTA	
OCN	F	GCTACCTTGGAGCCTCAGTC	113
	R	GGCGGTCTTCAAGCCATACT	
OSX	F	AGGCCTTTGCCAGTGCCTA	85
	R	GCCAGATGGAAGCTGTGAAGA	
Runx2	F	TGCAAGCAGTATTTACAACAGAGG	188
	R	GGCTCACGTCGCTCATCTT	
Wnt1	F	TGGGTTTCTACTACGTTGCTACTGG	117
	R	CGTCAACAGGTTCGTGGAG	
β-catenin	F	CCTAGCTGGTGGACTGCAGAA	137
	R	CACCACTGGCCAGAATGATGA	
LRP5	F	CACCATTGATTATGCCGACCAG	132
	R	TGAGTCAGGCCAAACGGGTAG	
OPG	F	CACACGAACTGCAGCACATT	188
	R	TCCACCAAAACACTCAGCCA	
RANKL	F	GCAGCATCGCTCTGTTCCTGTA	161
	R	CCTGCAGGAGTCAGGTAGTGTGTC	
RANK	F	ATCTCGGACGGTGTTGCAG	124
	R	TCTTCATTCCAGGTGTCCAAGTA	
GAPDH	F	AAATGGTGAAGGTCGGTGTGAAC	90
	R	CAACAATCTCCACTTTGCCACTG	

### Statistical analysis

Results were reported as mean ± SD and *P* < 0.05 was defined as the threshold of significance. Data were analyzed with the SPSS v 20.0.0 statistical software package (IBM, Chicago, IL, USA). For animal studies, body weights of time course study were analyzed by two-way repeated measures analysis of variance (ANOVA). The results were interpreted using the Greenhouse–Geisser correction to reduce the probability of obtaining a significant result by chance alone. Between subject factors consisted of intervention (Sham, OVX, OVX + LP, OVX + BP, OVX + HP and OVX + AP) and within subject factors consisted of time (weeks 0–8 after EMF stimulation) resulted in a 6 × 9 ANOVA. Data was analyzed for intervention and time main effects. Bonferroni-adjusted pairwise comparisons were performed for multiple comparisons of the means between the groups. EMF effect would be indicated by a significant main effect for intervention. One-way ANOVA (for normally distributed data) were used to analyze other dependent variables, and the Tukey post hoc test was performed for multiple comparisons among groups. For *in vitro* studies, each experiment contained a minimum of three replicates. Data presented are representative of at least three experiments. Data were analyzed using a one-way ANOVA followed by Tukey post hoc test for multiple comparisons in all experiments. For both *in vivo* and *in vitro* studies, the Kruskal-Wallis and Mann-Whitney U tests were performed when data were not normally distributed and the Bonferroni correction was utilized to correct for potential type I error that can occur when performing multiple comparisons.

## Results

### Effects of EMF on body weight

**Figure 4:**
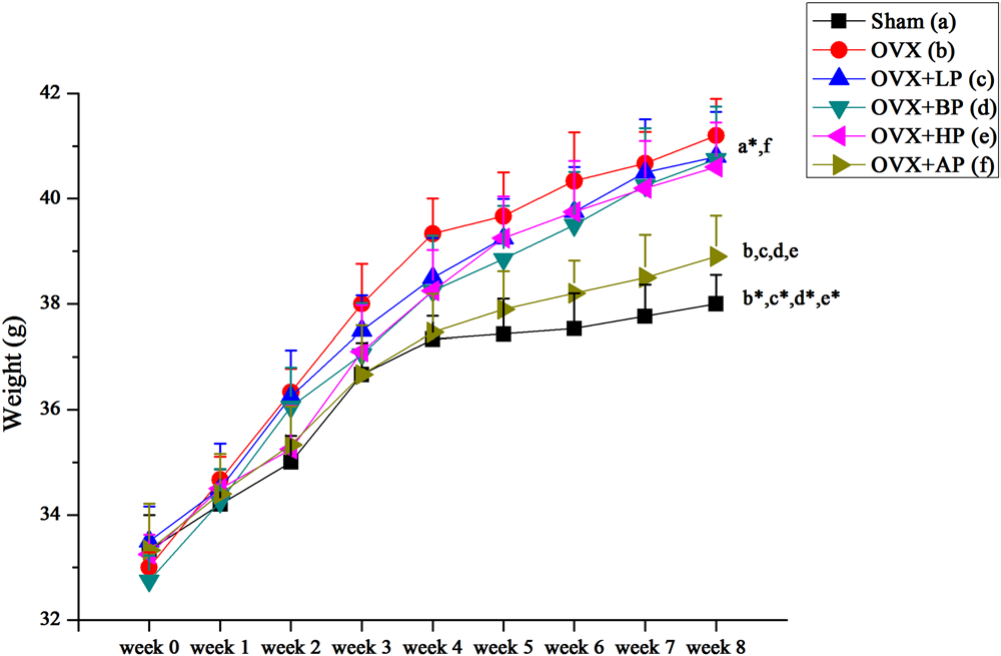
Trends of body weight in Sham, OVX, OVX + LP, OVX + BP, OVX + HP and OVX + AP groups at weeks 0–8 after EMF stimulation. Values represent mean ± SD of 8/group. Letters a-f indicate differences between respective groups at *P* < 0.05 or *P < 0.01 (a versus Sham; b versus OVX; c versus OVX + LP; d versus OVX + BP; e versus OVX + HP; f versus OVX + AP).

Two-way repeated measures ANOVA with a Greenhouse-Geisser correction determined that a significant main effect for time (F (2.209, 78.936) = 9.802, *P* < 0.001) was found for means of body weight throughout the time course (Fig. 4). The body weight differed significantly between time points. Post hoc tests using the Bonferroni correction revealed that OVX significantly increased the body weight of mice compared with Sham group (*P* < 0.01). No differences in body mass were present among LP, BP, HP and OVX (*P* > 0.05). AP reduced the body weight compared with OVX group (*P* < 0.05), with no difference over Sham groups.

### Biochemical analysis of serum

**Figure 5:**
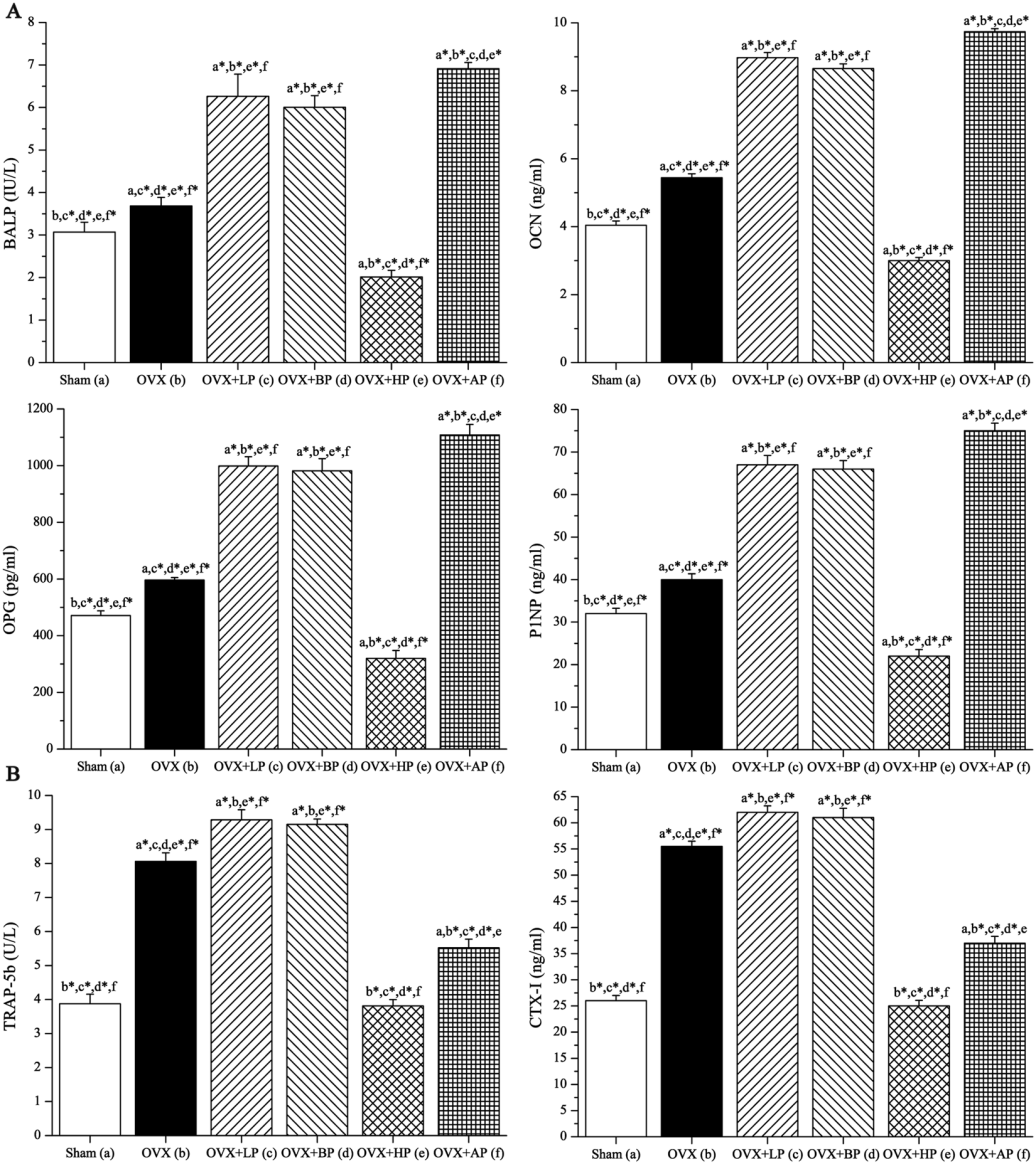
Effects of 8-week EMF exposure on serum biochemical indices (bone turnover markers) in OVX mice, including bone formation markers (**A**) and bone resorption markers (**B**). Values represent mean ± SD of 8/group. Letters a-f indicate differences between respective groups at *P* < 0.05 or *P < 0.01 (a versus Sham; b versus OVX; c versus OVX + LP; d versus OVX + BP; e versus OVX + HP; f versus OVX + AP).

As shown in Fig. 5, OVX led to increases in serum BALP, OCN, OPG and P1NP levels (bone formation markers) compared with Sham group (*P* < 0.05, + 20.1%, + 34.7%, + 26.6% and + 25.0%). After 8-week EMF interventions, LP and BP exposure similarly sharply elevated serum BALP, OCN, OPG and P1NP levels compared with OVX (*P* < 0.01, +70.0%, +64.8%, +67.5% and +67.5% for LP; *P* < 0.01, +63.0%, +59.0%, +64.7% and +65.0% for BP). AP also increased these bone formation markers (*P* < 0.01, +87.6%, +78.9%, +85.8% and +87.5%) compared with OVX, and the gain in AP was greater than LP and BP (*P* < 0.05). HP decreased serum BALP, OCN, OPG and P1NP compared with OVX (*P* < 0.01, −45.3%, −44.9%, −46.3% and −45.0%). What’s more, these bone formation markers in HP was lower than those in Sham (*P* < 0.05). In addition, OVX resulted in sharply increases in serum TRAP-5b and CTX-I (bone resorption markers) compared with Sham group (*P* < 0.01, +108.0% and +113.5%). After 8-week EMF interventions, serum TRAP-5b and CTX-I in LP and BP groups were higher compared with OVX (*P* < 0.05, +15.2% and +11.7% for LP; *P* < 0.05, +13.5% and +10.0% for BP). Serum TRAP-5b and CTX-I in HP were 52.7% and 55.0% respectively lower compared with OVX (*P* < 0.01), with no difference over Sham. In addition, serum TRAP-5b and CTX-I markers in AP were 31.5% and 33.3% respectively lower compared with OVX (*P* < 0.01), and slightly higher than those in Sham (*P* < 0.05).

### Bone mechanical characteristics

**Figure 6:**
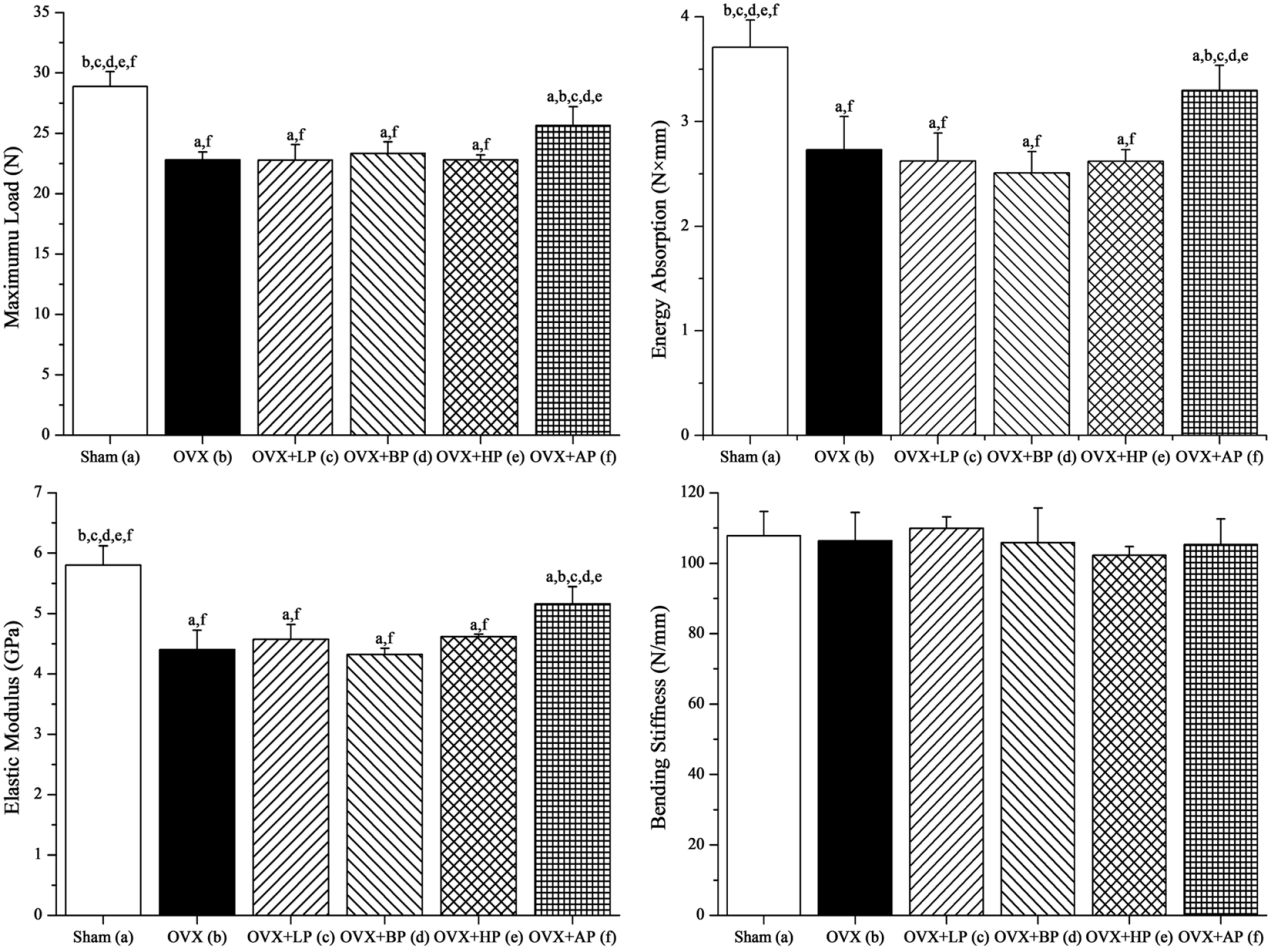
Effects of 8-week EMF exposure on femoral biomechanical structural properties in OVX mice via three-point bending test, including maximum load, energy absorption, elastic modulus and bending stiffness. Values represent mean ± SD of 8/group. Letters a-f indicate differences between respective groups at *P* < 0.05 or *P < 0.01 (a versus Sham; b versus OVX; c versus OVX + LP; d versus OVX + BP; e versus OVX + HP; f versus OVX + AP).

The three-point bending results of left femurs were shown in Fig. 6. OVX decreased maximum load, energy absorption and elastic modulus compared with Sham (*P* < 0.05, −21.1%, −26.4% and −24.2% respectively). After 8-week EMF interventions, no significant differences in these parameters were present among LP, BP, HP and OVX groups (*P* > 0.05). Bedsides, maximum load, energy absorption and elastic modulus in AP were 12.5%, and 20.8% and 17.3% (*P* < 0.05) respectively higher compared with OVX mice, and slightly lower than those in Sham (*P* < 0.05). In addition, no differences in bending stiffness were present among six groups (*P* > 0.05).

### µCT analysis of bone structure

**Figure 7:**
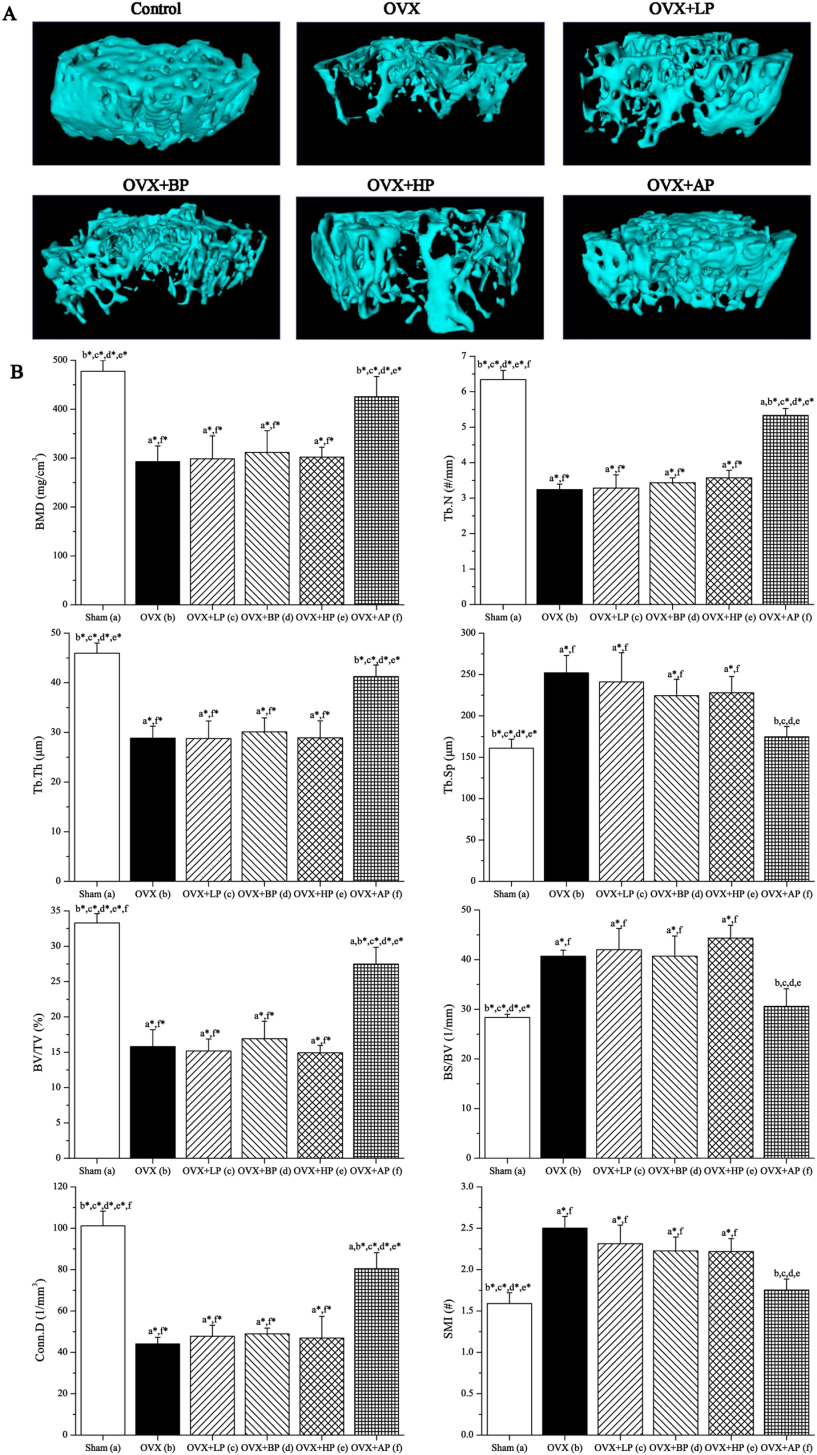
Effects of 8-week EMF exposure on trabecular bone microarchitecture in the distal femora in OVX mice. (**A**) Representative 3D µCT images of trabecular bone microarchitecture determined by the a volume of interest (VOI) that was 1 mm long 0.5 mm proximal to the growth plate. (**B**) Statistical comparisons of indices of trabecular bone microarchitecture. Values represent mean ± SD of 8/group. Letters a-f indicate differences between respective groups at *P* < 0.05 or *P < 0.01 (a versus Sham; b versus OVX; c versus OVX + LP; d versus OVX + BP; e versus OVX + HP; f versus OVX + AP).

**Figure 8:**
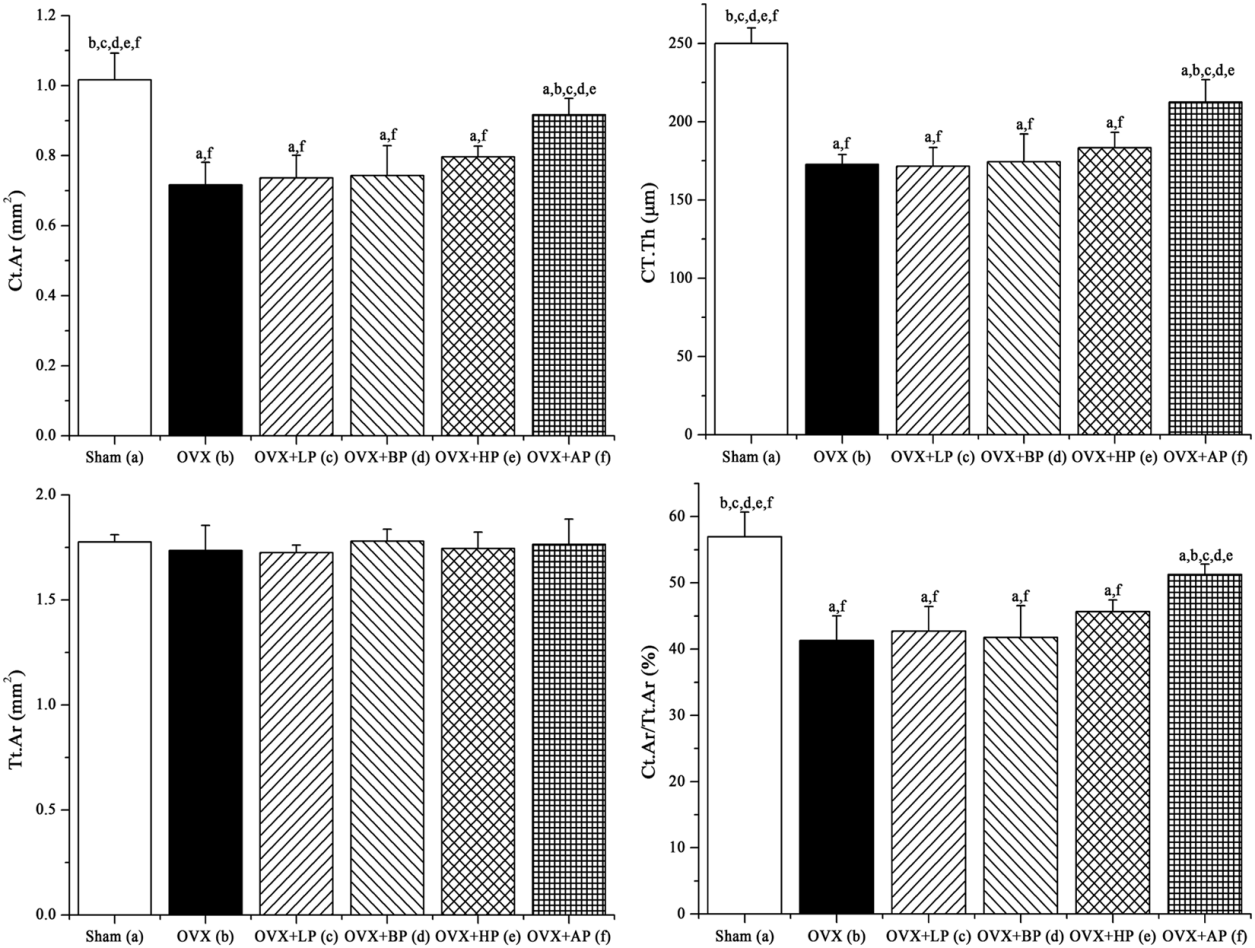
Effects of 8-week EMF exposure on cortical indices in the mid-diaphysis of the femur in OVX mice. Statistical comparisons of indices of trabecular bone microarchitecture. Values represent mean ± SD of 8/group. Letters a-f indicate differences between respective groups at *P* < 0.05 or *P < 0.01 (a versus Sham; b versus OVX; c versus OVX + LP; d versus OVX + BP; e versus OVX + HP; f versus OVX + AP).

Representative µCT mages for trabecular bone microarchitecture of left distal femur in six groups are shown in Fig. 7A. OVX resulted in notable reduction in trabecular bone microarchitecture. After 8-week EMF exposure, LP, BP and HP administration did not alter OVX-induced deterioration of trabecular bone microarchitecture. However, AP exposure significantly prevented trabecular bone loss. µCT analysis of the trabecular bone microarchitecture for left distal femur was presented in Fig. 7B, which indicated that OVX led to significant decreases in trabecular BMD, Tb.N, Tb.Th, BV/TV and Conn.D (*P* < 0.01, −38.7%, −48.9%, −37.2%, −52.5% and −56.4% respectively), and increases in Tb.Sp, BS/BV and SMI (*P* < 0.01, +56.7%, +43.5% and +57.3% respectively) compared with Sham group. After 8 week EMF exposure, no significant differences in these trabecular bone structural parameters were present among LP, BP, HP and OVX groups (*P* > 0.05). However, AP exposure significantly increased trabecular BMD, Tb.N, Tb.Th, BV/TV and Conn.D (*P* < 0.01, +45.3%, +64.5%, +43.0%, +73.8% and +82.4% respectively) compared with OVX. Besides, Tb.N, BV/TV and Conn.D in AP were slightly lower than those in Sham (*P* < 0.05), and no differences were present for BMD and Tb.Th compared with Sham (*P* > 0.05). Moreover, AP decreased Tb.Sp, BS/BV and SMI (*P* < 0.05, −30.7%, −24.8% and −29.9% respectively) compared with OVX group, and without difference compared with Sham (*P* > 0.05). Moreover, µCT analysis of the cortical bone parameters (Ct.Ar, Ct.Th, Tt.Ar and Ct.Ar/Tt.Ar) for mid-femur was presented in Fig. 8. OVX caused significant decreases in Ct.Ar, Ct.Th and Ct.Ar/Tt.Ar (*P* < 0.05, −29.5%, −30.9% and −27.8% respectively) as compared with the control group, but did not exert significant change in Tt.Ar (*P* < 0.05). After 8-week EMF interventions, no significant differences in these cortical bone parameters were present among LP, BP, HP and OVX groups (*P* > 0.05). However, Ct.Ar, Ct.Th and Ct.Ar/Tt.Ar in AP were 15.1%, and 16.0% and 11.3% (*P* < 0.05) respectively higher compared with OVX mice, and slightly lower than those in Sham (*P* < 0.05). In addition, no differences in Tt.Ar were present among six groups (*P* > 0.05).

### Histology and histomorphometry of trabecular bone

**Figure 9:**
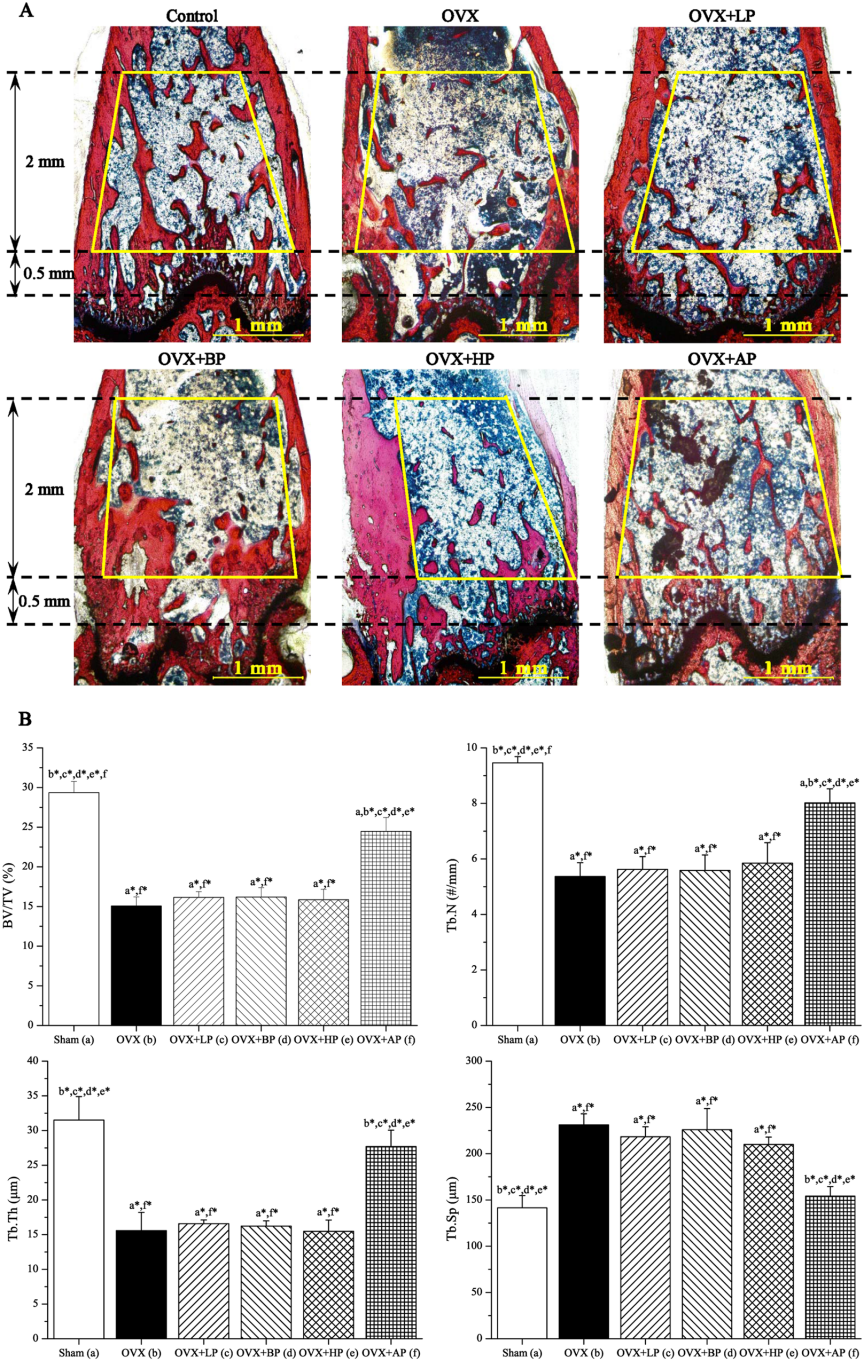
Effects of 8-week EMF exposure on trabecular bone histology and histomorphometry in OVX mice. (**A**) Representative histological images for bone microarchitecture of the distal femora by Van Gieson staining. Scale bar = 1 mm. (**B**) Histomorphometrical analysis of trabecular bone microarchitecture determined by an area of interest (AOI) that was 2 mm long 0.5 mm proximal to the growth plate. Values represent mean ± SD of 8/group. Letters a-f indicate differences between respective groups at *P* < 0.05 or *P < 0.01 (a versus Sham; b versus OVX; c versus OVX + LP; d versus OVX + BP; e versus OVX + HP; f versus OVX + AP).

Representative images by Van Gieson staining were shown in Fig. 9A. OVX in adult mice resulted in an almost complete ablation of trabecular bone in the left distal femur. Following 8 weeks, OVX significantly decreased BV/TV, Tb.N, Tb.Th (*P* < 0.01, −48.7%, −43.3% and −50.5%, respectively), and increased Tb.Sp (*P* < 0.01, +63.4%) (Fig. 9B). After 8-week EMF interventions, no significant differences were present among LP, BP, HP and OVX groups (*P* > 0.05). However, AP maintained significantly greater bone volume than OVX. BV/TV, Tb.N, Tb.Th in AP were 62.5%, 49.4% and 77.7% respectively higher than those in OVX (*P* < 0.01), and Tb.Sp in AP was 33.3% lower than that in OVX (*P* < 0.01). Besides, BV/TV and Tb.N were slightly lower than those in Sham (*P* < 0.05), and no differences were present for Tb.Th and Tb.Sp compared with Sham (*P* > 0.05).

### Real-time PCR

**Figure 10:**
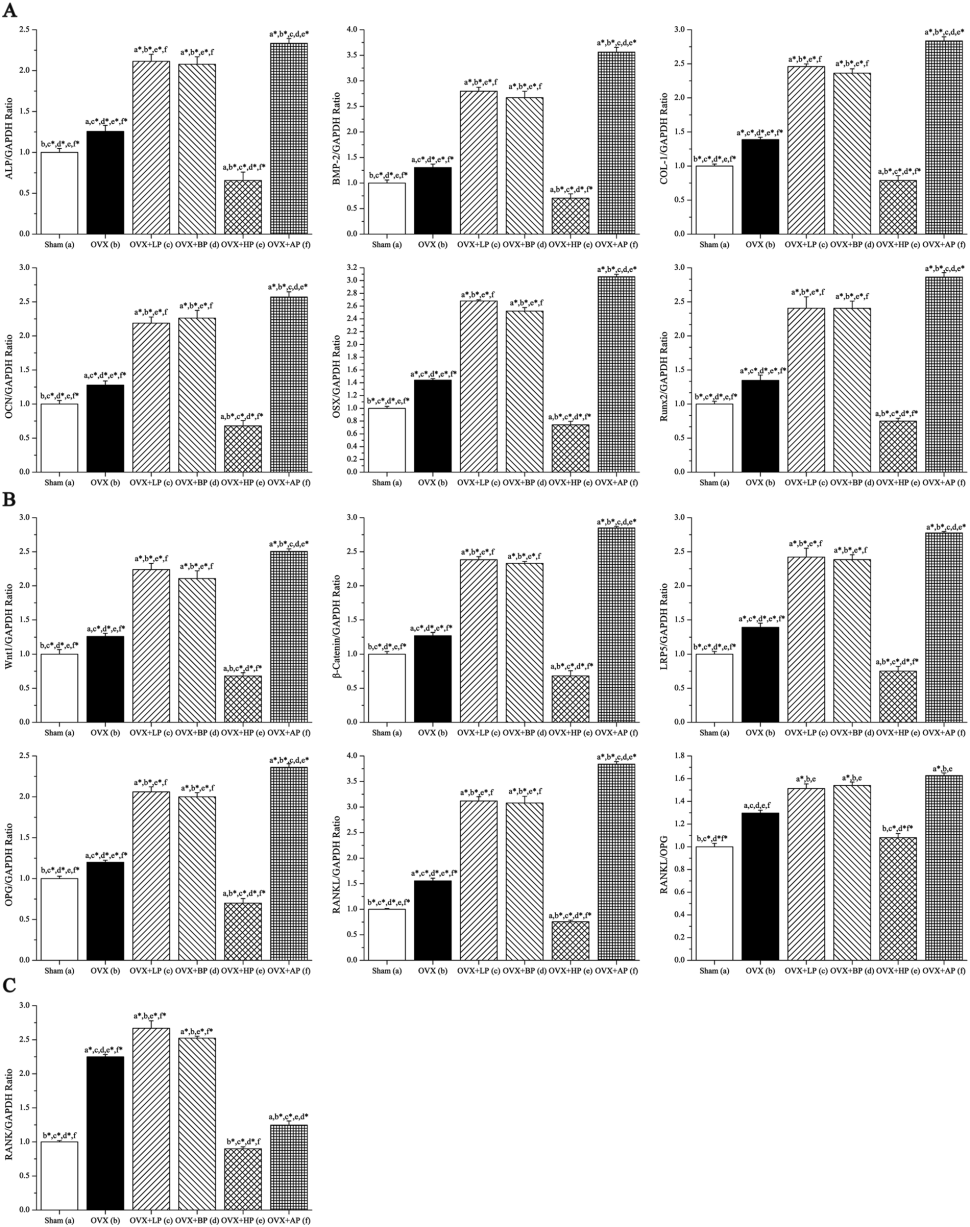
Effects of 8-week EMF exposure on gene expressions in the humerus with the removal of bone marrow in OVX mice by real-time fluorescence quantitative PCR analysis, including (**A**) osteogenesis-related gene expressions (**B**) Wnt1/β-catenin/LPR5 and OPG/RANKL signaling gene expression (**C**) osteoclastogenesis-related gene expression. Values represent mean ± SD of 8/group. Letters a-f indicate differences between respective groups at *P* < 0.05 or *P < 0.01 (a versus Sham; b versus OVX; c versus OVX + LP; d versus OVX + BP; e versus OVX + HP; f versus OVX + AP).

The results of real time PCR for total mRNA expressions in left humerus were shown in Fig. 10. ALP, BMP-2, COL-1, OCN, OSX and Runx2 are well known osteoblast differentiation and mineralization marker genes, and their mRNA expression levels in six groups after 8-week EMF exposure were presented in Fig. 10A. OVX increased the mRNA expression of ALP, BMP-2, OCN, COL-1, OSX and Runx2 compared with Sham (*P* < 0.05, +25.7%, +30.4%, +27.8%; *P* < 0.01, +38.7%, +44.1% and +34.8% respectively). The EMF with different frequency bands had different effects on gene expression levels. LP and BP sharply up-regulated the mRNA expression levels of ALP, BMP-2, COL-1, OCN, OSX and Runx2 compared with OVX (*P* < 0.01, +68.2%, +114.5%, +77.3%, +85.9%, +78.5% and +77.6% for LP; *P* < 0.01, +65.3%, +104.8%, +70.4%, +76.9%, +75.0% and +78.4% for BP respectively). The mRNA expression levels of ALP, BMP-2, COL-1, OCN, OSX and Runx2 in AP were 85.8%, 173.2%, 104.4%, 101.1%, 112.3% and 98.7% higher than those in OVX (*P* < 0.01). No difference was present between LP and BP (*P* > 0.05), and AP had more stimulative effect on the mRNA expressions for all osteogenesis-related genes than those in LP and BP (*P* < 0.05). In addition, HP significantly down-regulated the mRNA expression levels of ALP, BMP-2, COL-1, OCN, OSX and Runx2 compared with OVX (*P* < 0.01, −47.7%, −46.0%, −43.2%, −46.9%, −48.6% and −44.5% respectively). What’s more, these mRNA expression levels were slightly lower than those in Sham (*P* < 0.05).

Canonical Wnt1/β-catenin/LPR5 signaling plays a key role in mediating bone remodeling, and eventually regulates bone mass and bone strength. OPG and RANKL are cytokines predominantly secreted by osteoblasts and the relative concentration of RANKL and OPG (RANKL/OPG) play critical roles in bone mass and strength, and their mRNA expression levels in six groups after 8 weeks’ EMF exposure were presented in Fig. 10B. OVX increased the mRNA expression of Wnt1, β-catenin, OPG, LPR5, and RANKL compared with Sham (*P* < 0.05, +26.0%, +27.2%, +20.0%; *P* < 0.01, +39.1% and +55.7% respectively). LP and BP similarly progressively up-regulated the mRNA expression levels of Wnt1, β-catenin, LPR5, OPG and RANKL compared with OVX (*P* < 0.01, +77.6%, +87.3%, +74.1%, +71.7% and +100.3% for LP; *P* < 0.01, +67.2%, +83.2%, +71.4%, +66.7% and +97.9% for BP respectively). The mRNA expression levels of Wnt1, β-catenin, LPR5, OPG and RANKL in AP were 98.7%, 124.0%, 99.6%, 96.8% and 146.8% higher than those in OVX (*P* < 0.01). No difference was present among LP and BP (*P* > 0.05), and these mRNA expressions stimulated by AP were higher than those in LP and BP (*P* < 0.05). In addition, HP down-regulated the mRNA expression levels of Wnt1, β-catenin, LPR5, OPG and RANKL compared with OVX (*P* < 0.01, −46.0%, −46.4%, −46.0%, −41.7% and −51.4% respectively). What’s more, these mRNA expression levels were slightly lower than those in Sham (*P* < 0.05). In addition, OVX resulted in 29.7% increase in RANKL/OPG ratio compared with Sham (*P* < 0.05). LP, BP and AP stimulation similarly increased RANKL/OPG ratio, with relative expression values being 16.6%, 18.7% and 25.4% higher than that in OVX (*P* < 0.05), and no differences were present among these groups. HP exposure decreased RANKL/OPG ratio, with relative expression value being 18.7% lower than that in OVX (*P* < 0.05), and no difference was present between HP and Sham. It is known that RANK carry out important roles during osteoclast differentiation and activation. In our present study (Fig. 10C), OVX sharply increased the mRNA expression of RANK, with relative expression value being 124.9% higher than that in Sham (*P* < 0.01). LP and BP similarly up-regulated the mRNA expression levels of RANK compared with OVX (*P* < 0.05, +18.7% and +12.2% respectively). HP progressively down-regulated the mRNA expression levels of RANK compared with OVX (*P* < 0.01, −60.0%), with no difference over Sham. The RANK expression in AP was 44.5% lower than that in OVX, and slightly higher than that in Sham (*P* < 0.05).

## Discussion

The current study examined the effects of 8-week of four kinds of EMF with different frequency spectrum bands on OVX-induced OP in mice, and found that OVX resulted in the decrease of bone mass and deterioration of bone microarchitecture and mechanical strength in mice, and this condition were not altered by LP, BP and HP exposure. However, AP stimulation attenuated OVX-induced decrease of bone mass and deterioration of bone microarchitecture and mechanical strength in mice by promoting bone formation and inhibiting bone resorption.

Pulsed electromagnetic fields (PEMF) have been proven to present satisfying therapeutic effects on OP experimentally and clinically^9, 12, 37, 38^. However, the potential mechanism responsible for the effects of PEMF on preservation of bone mass are not well understood. PEMF carry a broad band of frequencies that occupy a discrete portion of the lower end of the electromagnetic spectrum. Although their repetition rate falls in the extremely low frequency range (1–100 Hz), their frequency contents in frequency domain by discreet Fourier transform ranges from 1 Hz to greater than 1 MHz^39, 40^. The present study explored the individual effects of different frequency components included in such broad band of frequencies on the bone formation and resorption in mice with OP. As a result, these combined effects might result in the therapeutic effects on bone mass and structure in OP.

Bone maintains its normal structural and functional integrity via continuous remodeling activity, characterized by a dynamic balance between OBs-mediated bone formation and OCs-mediated bone resorption. Estrogen deficiency induced by OVX results in bone loss due to an accelerated rate of bone resorption which predominates over bone formation^41, 42^. In our present study, the bone formation activity was slightly enhanced in OVX mice, which was demonstrated by slightly increased serum bone formation markers (BALP, OCN, OPG and P1NP) and osteogenesis-related gene expressions (ALP, BMP-2, COL-1, OCN, OSX, Runx2, Wnt1, β-catenin, LPR5 and OPG) compared with Sham. Besides, the bone resorption activity was significantly enhanced in OVX mice, with predominance over bone formation, which was demonstrated by increased serum bone resorption markers (TRAP-5b and CTX-I) and osteoclastogenesis-related gene expression (RANK and RANKL/OPG) compared with Sham. As a result, accelerated bone remodeling and perturbation in bone mineral homeostasis might led to decrease of bone mass and deterioration of bone microarchitecture and mechanical strength in OVX mice, which was demonstrated by increase in body weight, decreased biomechanical, µCT and histological characteristics of bone in mice 8 weeks after OVX. These results were also reported by numerous investigators^11, 13, 29, 30, 38^.

In addition, LP and BP were similarly capable of significantly increasing bone formation activity in OVX mice, which was demonstrated by increased serum bone formation markers (BALP, OCN, OPG and P1NP) and osteogenesis-related gene expressions (ALP, BMP-2, COL-1, OCN, OSX, Runx2, Wnt1, β-catenin, LPR5 and OPG) compared with OVX. The increased bone formation activity comes from anabolic functional responses of OBs (Supporting Figs S2 and S3), which might be induced by resonant effectiveness of numerous Ca^2+^ according to ICR model^22–24^. The time-varying EMF in proper resonance might transfer kinetic energy to channel ions^23^. This will increase ionic drift velocities through the membrane, easily activating the voltage-dependent L-type Ca^2+^ channels and increasing intracellular calcium signals, and downstream responses of increased Ca^2+^ might be mediated through Ca^2+^/nitric oxide/cGMP/protein kinase G pathway^43^. Potentially, therapeutic responses may be largely as a result of nitric oxide/cGMP/protein kinase G pathway stimulation^43^. Bedsides, LP and BP also slightly increased the bone resorption activity compared with OVX, evidenced by the increased RANKL/OPG mRNA ratio brought by increased osteoblastic activity. However, LP and BP did not directly regulate osteoclastogenesis-related gene expression which was demonstrated by our *in vitro* results (Supporting Fig. S4). Although resonant effectiveness of numerous Ca^2+^ also exists in OCs, the voltage-dependent L-type Ca^2+^ channels in OCs might not be activated sensitively^44, 45^. To view the situation as a whole, the bone resorption activity in OVX + LP and OVX + BP weights than bone formation. Thus, LP and BP exposure did not ameliorate decrease of bone mass and deterioration of bone microarchitecture and mechanical strength in OVX mice.

HP not only sharply decreased the bone formation activity but also progressively decreased bone resorption activity compared with OVX, evidenced by decreased serum bone formation and resporption markers and osteogenesis and osteoclastogenesis related gene expressions compared with OVX. Although high frequency magnetic field could not ignite resonate effect, the electric field with high frequency could be induced. Thus, the cytoplasm is penetrated by a high frequency electric field^46^, which might lead endoplasmic reticulum and mitochondria to release large amount of calcium into cytoplasm^47, 48^. As a result, disturbance of anabolic functional response and initiation event for apoptosis could be brought about^49, 50^. Although bone resorption activity was inhibited by HP exposure, the bone formation activity was also inhibited by HP exposure. Therefore, HP might not ameliorate decrease of bone mass and deterioration of bone microarchitecture and mechanical strength induced by OVX in mice.

AP might have accumulative effects of LP, BP and HP, which significantly increased bone formation and decreased bone resporption compared with OVX. This view was demonstrated by increased serum bone formation and osteogenesis-related gene expressions and decreased bone resporption markers and osteoclastogenesis-related gene expressions compared with OVX. As a result, AP exposure ameliorated decrease of bone mass and deterioration of bone microarchitecture and mechanical strength induced by OVX in mice, which was demonstrated by decreased body weight, increased biomechanical, µCT and histological characteristics of bone in mice 8 weeks after OVX. To our knowledge, no investigations have directly reported the same results exposured by the same EMF as in our study. However, similar results were found by using EMF whose frequency spectrum in frequency domain include both ICR frequency and high frequency components. 15 Hz PEMF with 200 µs pulse width exposure presented stimulus efficacy in OVX-induced bone loss in rats^51^. According to our analysis, the frequency spectrum of 15 Hz PEMF with 200 µs pulse width in frequency domain lies in the bandwidth from 15 to 5,000 Hz in frequency domain. Therefore, ICR frequency and high frequency components are all involved in this kind of PEMF. Consistently, 8 Hz PEMF with 200 µs pulse width (band width of frequency spectrum: 8–5,000 Hz) stimulation also prevent OVX-induced OP in rats^13, 38, 52^.

EMF has been a non-pharmacological and non-invasive alternative method for the treatment of osteoarthritis, brain and cardiac ischemia and traumatic brain injury^53^. However, epidemiology studies reported that EMF might in relation to brain cancer and leukemia^54–56^. Despite many *in vitro* and *in vivo* investigations, there is no established causal relationship yet. The inconsistencies on the effects of EMF might come from different type of EMF employed in both laboratory research and clinical trial. According to our experiment, the low frequency EMF might have positive effects on OBs, and the high frequency EMF might have side effects on OCs. Moreover, this side effects of high frequency EMF is required for the treatment of osteoporosis. What’s more, the side effects of high frequency EMF might also be useful to suppress abnormal cells such as cancer cells. However, it should be cautious to use EMF devices clinically and EMF devices cannot be recommended without scientific evidence from high-quality, double-blind and randomized trial.

In summary, our results showed that ICR related frequency components significantly increased bone formation activity, and it slightly increased bone resorption activity indirectly. However, predominated bone resorption activity resulted in limited effects on decrease of bone mass and deterioration of bone microarchitecture and mechanical strength in OVX-induced OP in mice. Besides, high frequency components not only sharply decreased the bone formation activity but also progressively decreased bone resorption activity. As a result, HP had limited effects on decrease of bone mass and deterioration of bone microarchitecture and mechanical strength in OVX-induced OP in mice. What’s more, combined ICR related frequency with high frequency components could have therapeutic effects on bone loss in OVX-induced OP in mice, which results from increased bone formation and decreased bone resporption.

## Electronic supplementary material

Supplementary Information
