# Supplementary material for: Effects of four kinds of electromagnetic fields (EMF) with different frequency spectrum bands on ovariectomized osteoporosis in mice
Source: Sci Rep. 2017 Apr 3;7:553. doi: 10.1038/s41598-017-00668-w (PMC5428825; doi:10.1038/s41598-017-00668-w)
Supplement: Supplementary file 1 — Supplementary Information [file 41598_2017_668_MOESM1_ESM.doc]

**Effects of four kinds of time-varying electromagnetic fields (EMFs) with different frequency spectrum bands on osteoblasts and osteoclasts in vitro**

Tao Lei^1,*^, Feijiang Li^1,*^, Zhuowen Liang^2,*^, Chi Tang^1^, Kangning Xie^1^, Pan Wang^1^, Xu Dong^1^, Shuai Shan^1^, Juan Liu^1^, Qiaoling Xu^3^, Erping Luo^1^ & Guanghao Shen^1^

**1** School of Biomedical Engineering, Fourth Military Medical University, 17 West Changle Road, Xi’an, China,

**2** Institute of Orthopaedics, Xijing hospital, Fourth Military Medical University, Xi'an, China

**3** School of Nursing, Fourth Military Medical University, 17 West Changle Road, Xi’an, China

*These authors contributed equally to this work

Correspondence and requests for materials should be addressed to E.L. (email: erpingluo@hotmail.com) or G.S. (email: [guanghaoshen@outlook.com](mailto:guanghaoshen@outlook.com))

**Materials and Methods**

In vitro exposure procedures

For OBs, the osteoblast-like cell line MC3T3-E1 subclone 4 was purchased from ATCC. Five groups including Control, LP, BP, HP and AP were employed in the experiments. LP, BP, HP and AP groups were stimulated with four kinds of different EMFs three hours every day for six days. For OCs, mouse monocyte RAW 264.7 macrophage-like were purchased from the ATCC. Six groups including Control, RL (RANKL), RL+LP, RL+BP, RL+HP and RL+AP were used in the experiments. After the cells were plated into culture plates and allowed to confluence, 50 ng/ml RANKL (PeproTech, Rocky Hill, NJ, USA) was added to five groups with the exception of Control group. Then, four groups were stimulated with four kinds of different EMFs three hours every day for six days, except for the Control and RL group. Osteogenesis and osteoclastogenesis-related proteins were examined using western bolt.

After EMFs exposure, total protein (estimation for alkaline phosphatase (ALP), bone morphogenetic protein-2 (BMP-2), type 1 collagen (COL-1), osteocalcin (OCN), osterix transcription factors (OSX), runt-related transcription factor 2 (Runx2), Wnt1, β-catenin, low-density lipoprotein receptor-related protein 5 (LRP5), osteoprotegerin (OPG) protein levels) and crude membrane protein fractions (estimation for RANKL and RANK protein levels) were prepared from cultured cells. For total protein extraction, cells were washed with ice-cold PBS, and 100 µl sassay lysis buffer (RIPA) containing phenylmethylsulfonyl fluoride (PMSF) (RIPA : PMSF, 100 : 1, v : v) (Membrane and Cytosol Protein Extraction Kit, Beyotime Institute of Biotechnology, Shanghai, China) was added to each well and kept for 30 min on ice. The cell lysate was collected and centrifuged at 12,000 rpm for 10 min at 4°C. The supernatant was collected in a new microcentrifuge tube as total protein. The collection method for rude membrane protein fractions referenced as JAY CAO et al. The total protein and membrane protein were mixed with 5X loading buffer (loading buffer: supernatant, 1:4, v:v) and loaded onto a 12% sodium dodecyl sulfate (SDS) polyacrylamide gel. Electrophoresis was performed to transfer separated protein onto a polyvinylidene fluoride membrane (PVDF, Thermo Fisher Scientific, Waltham, MA, USA). After blocking with phosphate-buffered saline (PBS) containing 5% skim milk for 1 h at room temperature, the membrane was incubated in diluted primary antibody solution at 4°C overnight without shaking. After three rinses with 0.05% Tween-20 in PBS (v/v) (PBST), the PVDF membrane was incubated with diluted secondary antibody solution (conjugated to horseradish peroxidase). Then, enhanced chemiluminescence (ECL) substrate (Thermo Scientific, USA) was applied to incubate the membrane. Target bands were detected by standard X-ray films and CCD camera (Advanced Molecular Vision). Densitometry analysis of target bands was semi-quantified using Image-Pro Plus (Media Cybernetics) for statistical analysis. Beta-actin was used to normalize the cellular protein content. The results presented were from three separate experiments.

**Results**

In vitro osteogenesis-related protein expessions in OBs

Results of in vitro osteogenesis-related protein expressions after 6-day EMF exposure (3 h/day) including ALP, BMP-2, COL-1, OCN, OSX and Runx2 via Western blot analysis were shown in Supporting Fig. S1. The Western blot results showed that the protein expressions of ALP, BMP-2, COL-1, OCN, OSX and Runx2 were significantly higher in LP, BP and AP than those in Control (*P* < 0.01, +60.2%, +78.0%, +131.5%, +69.2%, +169.6% and +53.3% for LP; *P* < 0.01, +46.0%, +103.3%, +126.3%, +56.0%, +154.0% and +64.7% for BP; *P* < 0.01, +86.2%, +126.6%, +161.5%, +85.2%, +198.7% and +83.4% for AP). No difference was present between LP and BP (*P* > 0.05), and AP had more stimulative effects on these protein expressions than those in LP and BP (*P* < 0.05). In addition, HP significantly decreased the protein expressions of ALP, BMP-2, COL-1, OCN, OSX and Runx2 compared with Control (*P* < 0.01, -50.0%, -54.7%, -36.3%, -40.0%, -34.0% and -34.5% respectivly), which indicated that HP exposure decreased the protein expressions of ALP, BMP-2, COL-1, OCN, OSX and Runx2.

Protein expressions of Wnt1, β-catenin, LPR5, OPG, RANKL and RANKL/OPG were presented in Fig. 8. The protein expressions of Wnt1, β-catenin, LPR5, OPG and RANKL were higher in LP, BP and AP than those in Control (*P* < 0.01, +55.2%, +67.5%, +144.6%, +42.1% and +88.4% for LP; *P* < 0.01, +44.3%, +62.6%, +130.0%, +40.0% and +71.9% for BP; *P* < 0.01, +63.1%, +69.4%, +171.4%, +57.9% and +113.0% for AP). No difference was present between LP and BP (*P* > 0.05), and AP had more stimulative effects on these protein expressions than those in LP and BP (*P* < 0.05). HP significantly decreased Wnt1, β-catenin, LPR5, OPG and RANKL (*P* < 0.01, -36.5%, -60.7%, -44.4%, -33.8% and -47.3% respectively). In addition, RANKL/OPG protein ratio in LP, BP and AP were 32.5%, 23.1% and 35.0% respectively higher than that in Control (*P* < 0.05), and no significant differences were present among these groups. HP decreased RANKL/OPG protein ratio compared with Control, with relative expression value being 20.3% lower than that in Control (*P <* 0.05).

In vitro osteoclastogenesis-related gene expressions in OCs

Results of in vitro osteoclastogenesis-related protein expression (RANK) after 6 days’ EMFs exposure (3 h/day) was shown in Supporting Fig. S4. The Western blot results showed that no RANK protein was expressed in Control group. Six days’ RANKL administration induced formation of mature OCs, thus RANK expressed in RANKL group. LP and BP stimulation did not alter RANK expression compared with RANKL group (*P* > 0.05), and no difference was present between LP and BP. Besides, HP and AP significantly decreased RANK expression compared with OVX group (*P* < 0.01), and no difference was present between HP and AP.

**Figure S1**

**
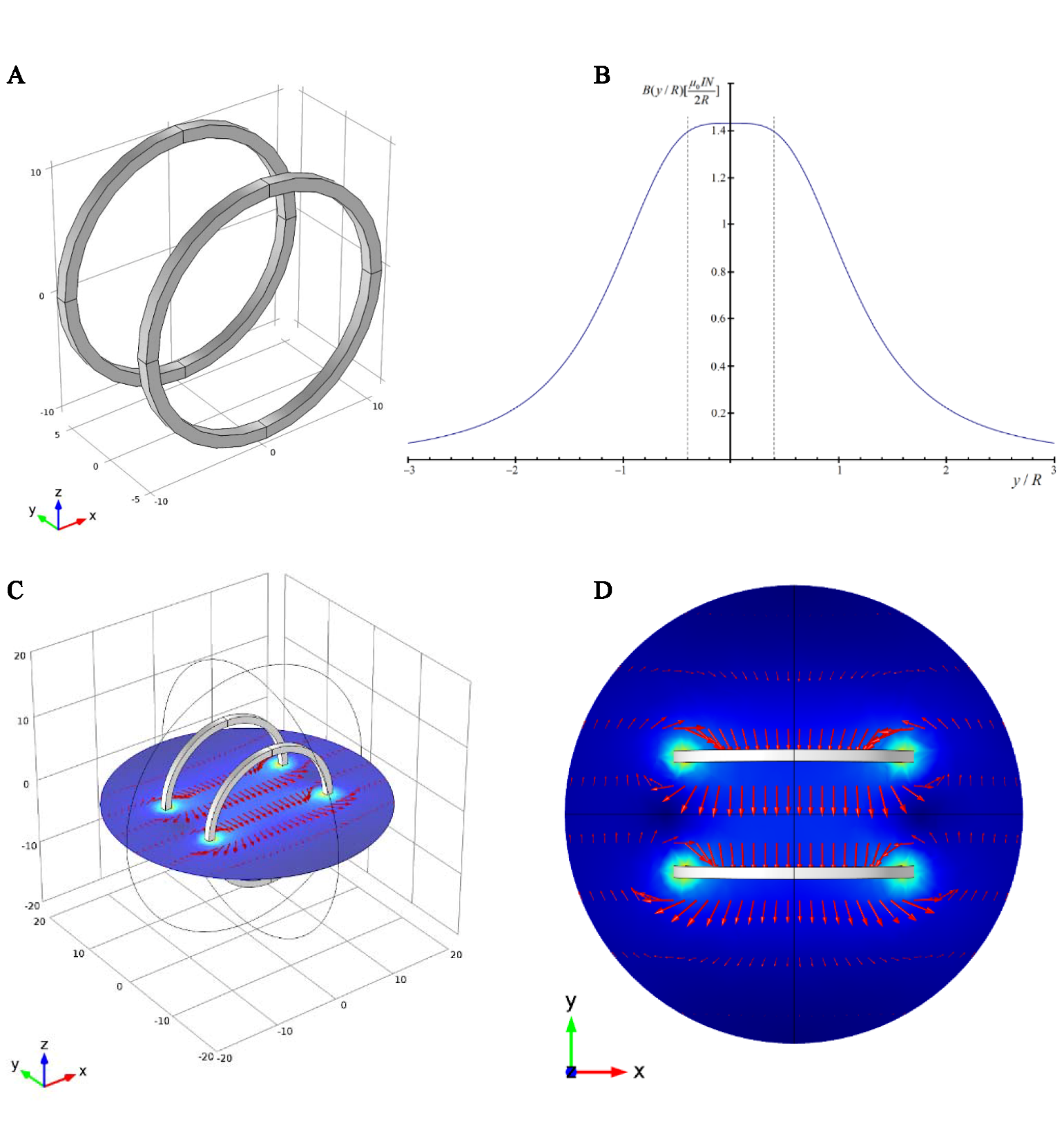
**

**Fig. S1.** The demonstration of the uniformity of the EMFs. (A) The geometric model of Helmholtz coil established by the ﬁnite element engineering software called COMSOL Multiphysics. (B) Magnetic flux density along the axis crossing the center of coils at XY plane, y = 0 is the point in the middle of distance between coils. (C) Three dimensional distribution of the magnetic flux density in the Helmholtz coils at XY plane when the current in the coils is a constant value, whose homogeneous color indicates the peak-to-peak magnetic flux density at XY plane was uniform. (D) Two dimensional distribution of magnetic flux density in the Helmholtz coils at XY plane**.**

**Figure S2**

**
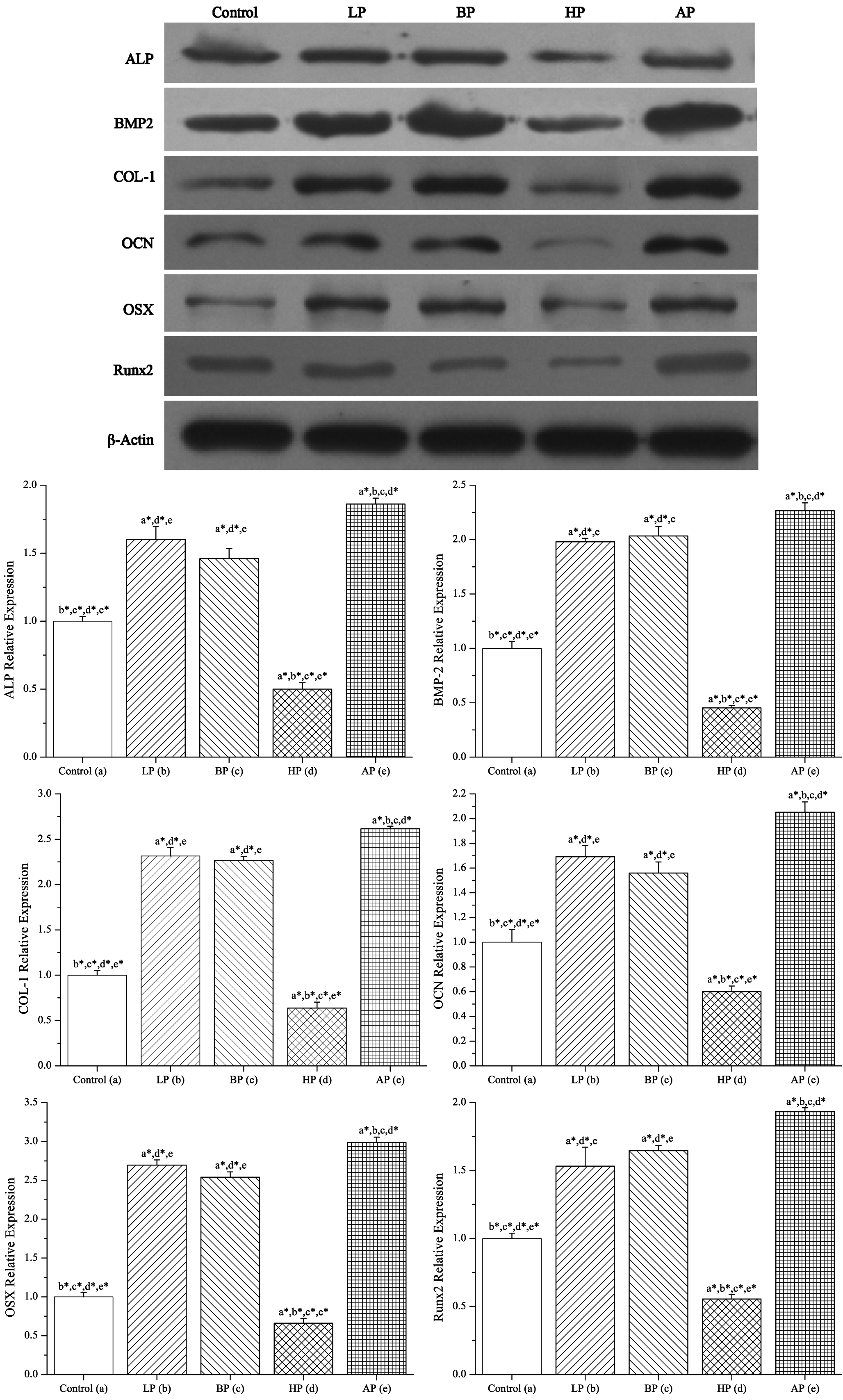
**

**Fig. S2.** Protein expression levels of ALP, BMP-2, COL-1, OCN, OSX and Runx2 in MC3T3-E1 cells after stimulation with EMFs for 8 days. Values represent mean ± SD of 3/group. Letters a-e indicate differences between respective groups at *P* < 0.05 or **P* < 0.01 (a versus Control; b versus LP; c versus BP; d versus HP; e versus AP).

**Figure S3**

**
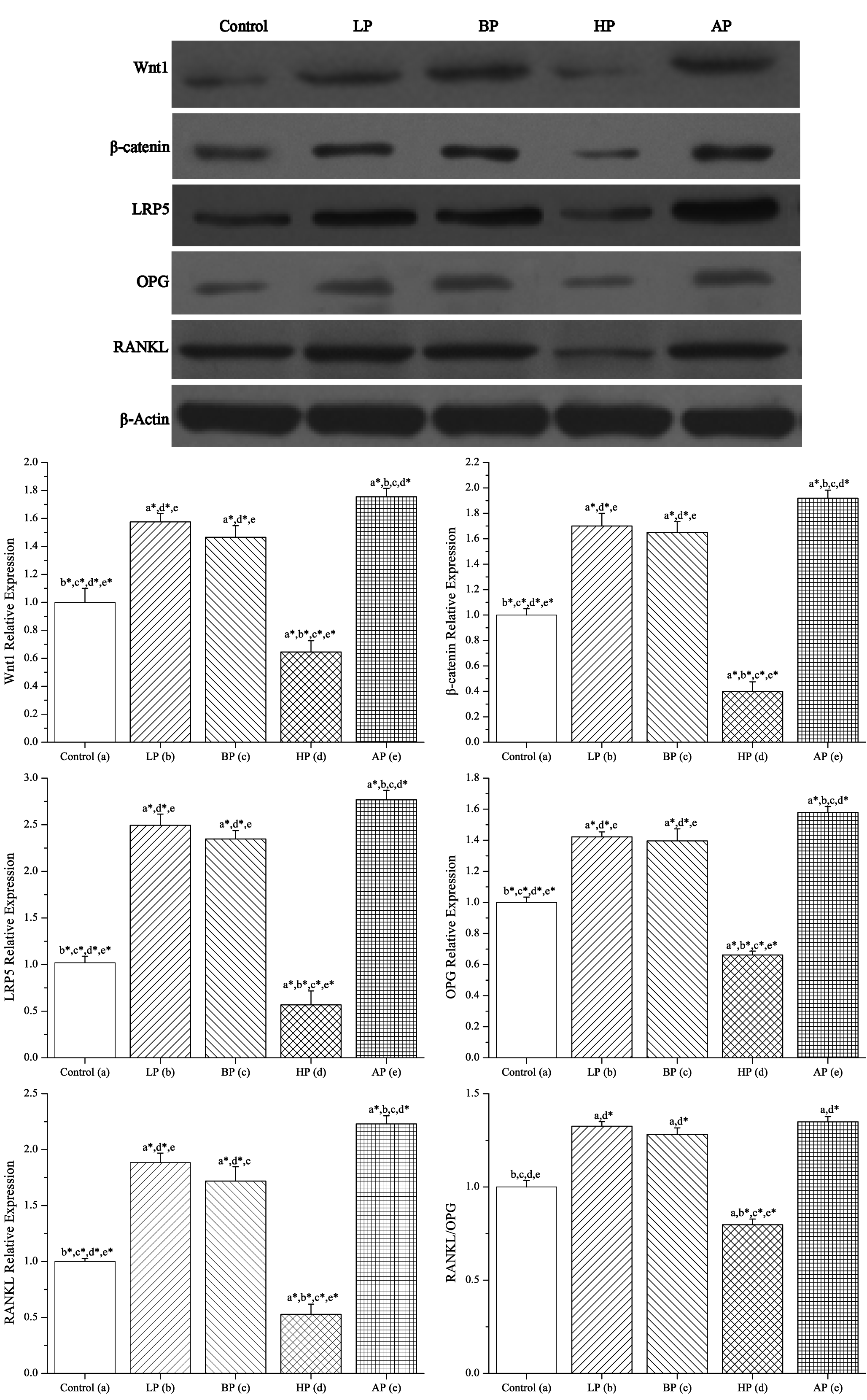
**

**Fig. S3.** Protein expression levels of Wnt1, β-catenin, LPR5, OPG, RANKL and RANKL/OPG in MC3T3-E1 cells after stimulation with EMFs for 6 days. Values represent mean ± SD of 3/group. Letters a-e indicate differences between respective groups at *P* < 0.05 or **P* < 0.01 (a versus Control; b versus LP; c versus BP; d versus HP; e versus AP).

**Figure S4**

**
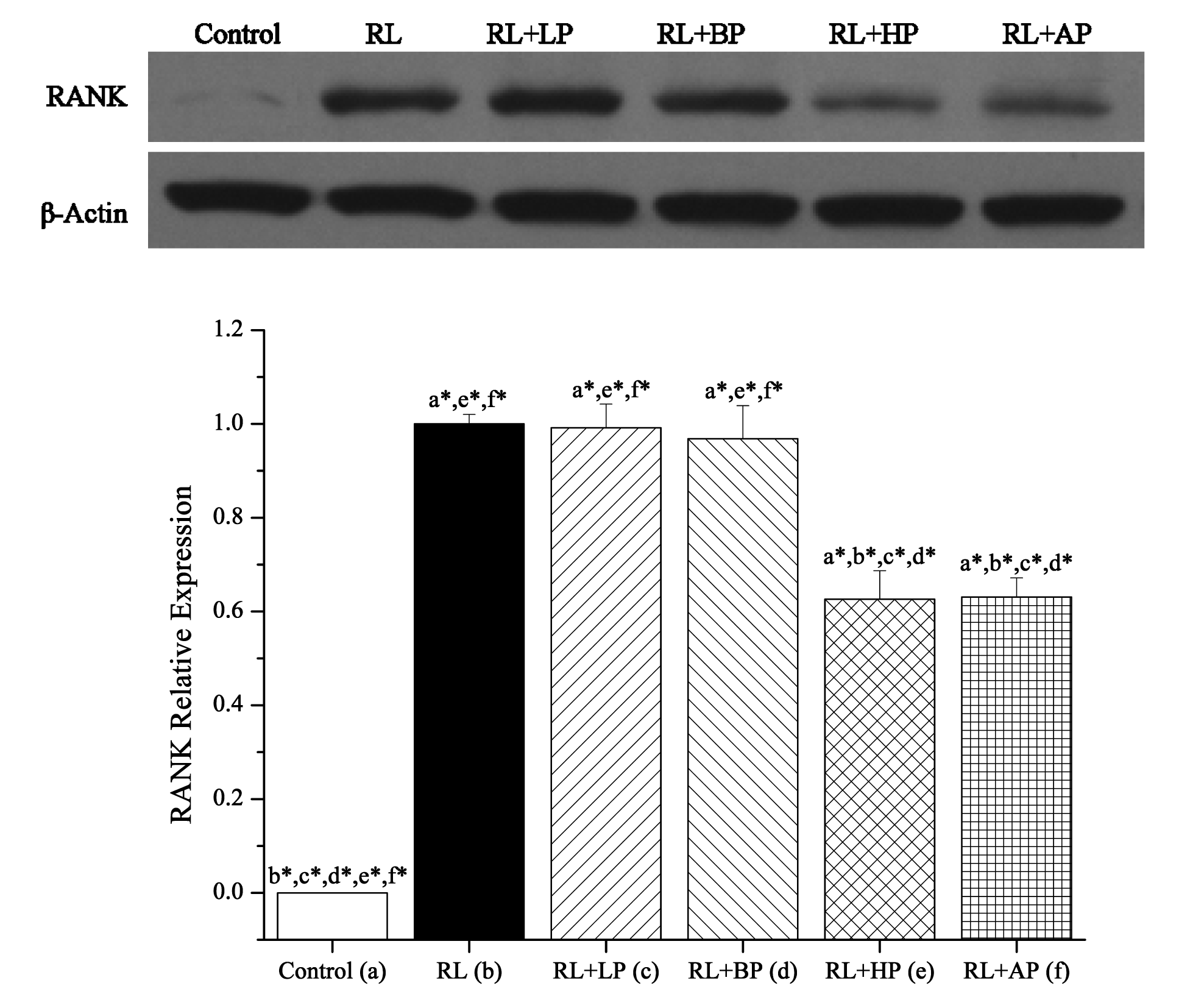
**

**Fig. S4.** Protein expression levels of RANK in RANKL induced RAW 284.7 cells after stimulation with EMFs for 6 days. Values represent mean ± SD of 3/group. Letters a-f indicate differences between respective groups at *P* < 0.05 or **P* < 0.01 (a versus Control; b versus RL; c versus RL+LP; d versus RL+BP; e versus RL+HP; f versus RL+AP).

**Supplemental Information References**

1. Cao J, Venton L, Sakata T, Halloran BP. Expression of RANKL and OPG correlates with age-related bone loss in male C57BL/6 mice. J Bone Miner Res. 2003;18(2):270-7.

2. Yang HK, Jeong KC, Kim YK, Jung ST. Role of matrix metalloproteinase (MMP) 2 and MMP-9 in soft tissue sarcoma. Clinics in orthopedic surgery. 2014;6(4):443-54.
